# Perspective of Clean Energy-saving by Semiconducting Quantum Dot Nanomaterials through Photoelectric and Density of States Analysis

**DOI:** 10.1007/s10895-025-04207-z

**Published:** 2025-02-20

**Authors:** Fatemeh Mollaamin, Majid Monajjemi

**Affiliations:** 1 Department of Biomedical Engineering, Faculty of Engineering and Architecture, Kastamonu University, Kastamonu, Turkey; 2 Department of Chemical Engineering, Central Tehran Branch, Islamic Azad University, Tehran, Iran

**Keywords:** Clean energy, Na/K-ion battery, Energy storage, Density of states, Hydrogen adsorption

## Abstract

With the pressure for renewable energy resources and the enchantingly digitalized current lifestyle, the need for batteries will augment. Therefore, in this article, it has been evaluated the promising alternative alkali metals of sodium-ion and potassium-ion, batteries. The hypothesis of the hydrogen adsorption phenomenon was confirmed by density distributions of charge density differences (CDD), total density of state (TDOS), and electron localization function (ELF) for of Li[GeO–SiO], Na[GeO–SiO] or K[GeO–SiO] heterostructures that have revealed an efficient charge transfer owing to the internal electric field. Regardless of adsorption configurations of H_2_ molecules, the region of charge density variation is mainly concentrated between the H_2_ molecule and the layers of Li[GeO–SiO], Na[GeO–SiO] or K[GeO–SiO] heterostructures atoms. The maximum energy of TDOS for K[GeO–SiO] with several peaks around –0.35, –0.45, –0.6 and –0.75 a.u. with maximum density of state of ≈ 23 around –0.35 a.u. has been revealed. As the advantages of lithium, sodium or potassium over Si/Ge possess its higher electron and hole motion, allowing lithium, sodium or potassium instruments to operate at higher frequencies than Si/Ge instruments. K[GeO–SiO]–2H_2_ and Na[GeO–SiO]–2H_2_ heterostructures with band gap of 0.9230 and 0.8963 eV, respectively can be more efficient for hydrogen grabbing. The findings suggest that the proposed heterostructures offer appropriate band edge positions for saving energy in the batteries. Furthermore, the calculations have revealed that non-magnetic dopants can induce stable half-metallic ferromagnetic ground state in Li/Na/K. In particular, at the same levels of doping, the K/Na-doped [GeO–SiO] heterostructure framework exhibited the strongest H_2_ binding.

## Introduction

Nanomaterials with remarkable specific structures indicate promising applications in the field of energy storage, electrocatalysis, and fuel cells. Although lithium-ion batteries have become available in the current technology perspective through their high energy density, they face serious discussions in terms of safety and sustainability [1–6]. Recently, the findings of an investigation illustrated high specific capacity, low Li diffusion energy barrier, and low open circuit voltage for the Mn_2_O_3_-based anode for use in LIBs [7].

Along with the sodium ion, potassium-ion is the prime chemistry replacement candidate for lithium-ion batteries [8–14]. One research provided theoretical insights into the potential uses of boron-nitride nanotubes and beryllium-oxide nanotubes as anodes in Na, K, and lithium-ion batteries [15].

The researchers investigated exothermic interactions of Li, Na, Mg, Ca, K, and Zn with the host SrRuO_3_ which show their appropriateness for the intercalation process in the batteries. The findings of the study predict the excellent anodic performance of SrRuO_3_ for multivalent ion batteries [16]. Another research demonstrated the prospects of In_2_CO slab for utilization as promising material for hydrogen storage through applying electric field to fully loaded slab [17].

Recently, scientists have reported the structural, electronic, thermal, and optical properties of a novel material In_2_CO in bulk phase and slab models in (001), (100), (101), and (011) orientations. The direction-dependent study of optical characteristics revealed that the (001) slab of the material can be an efficient infrared detector [18].

Recently, a scientific research tried to improve the performance of Magnesium ion batteries as substitution of lithium-ion batteries for energy storage devices. A theoretical study was conducted to examine the potential of Al_2_CO bilayer as an anode material in magnesium ion batteries [19].

Recently, Si-, Ge- or Sn-carbide nanostructures have been suggested as engaged H-grabbing compounds [20–22]. Since the polarizability of silicon is more than carbon, it is supposed that Si–C/Si nanosheet might attach to compositions more strongly in comparison to the net carbon nano-surfaces [23–26]. As Na-ion batteries are an alternative to the Li-ion batteries owing to the expand availability of sodium, its low cost, and nontoxicity, the researchers investigated the Na and Na^+^ adsorption on nanosheets of carbon, AlN, BN, and SiC to unravel their potential use as an anode in NIBs [27].

In our previous works, the investigation of energy storage in fuel cells through hydrogen adsorption has been accomplished using DFT calculations through different nanomaterials consisting of silicon/germanium/tin/lead nano-carbides [28], magnesium -aluminum alloy [29] and aluminum/carbon/ silicon doping boron nitride nanocage [30]. Currently, the present research aims to explore the possibility of using Li[GeO–SiO], Na[GeO–SiO] or K[GeO–SiO] nanocluster for hydrogen storage by employing first-principles calculations. We have analyzed the structural and electronic properties of Li[GeO–SiO], Na[GeO–SiO] or K[GeO–SiO] nanocluster and hydrogenated nanostructures of Li[GeO–SiO]–2H_2_, Na[GeO–SiO]–2H_2_ or K[GeO–SiO]–2H_2_ using state-of-the-art computational techniques. Today, it is crucial to distinguish the potential of hydrogen technologies and bring up all perspectives of their performance, from technological progresses to economic and social effects. The authors intend to pursue research on sustainability and clean energy subjects towards finding new solutions for reducing the global dependency on fossil fuels.

## Theoretical Background, Materials & Approaches

The first principles calculations assess an important function in developing and optimizing new energy saving and conversion materials [31].

In DFT, as it is used for computational chemistry, the hybrid functional Becke 3-parameter Lee–Yang–Parr (B3LYP) [32] appears to offer the greatest contribution. A new hybrid exchange–correlation functional named Coulomb-Attenuating Method with B3LYP (CAM-B3LYP) is proposed which combines the hybrid qualities of B3LYP and the long-range correction [33].

Besides, In the DFT–D3 method of Grimme et al., the following expression for the Van Der Waals (vdW)-dispersion energy-correction term is used [34]:

1 $${E}_{\text{disp}}= -\frac{1}{2} \sum\nolimits_{i=1}^{{N}_{\alpha t}}\sum\nolimits_{j=1}^{{N}_{\alpha t}}\sum\nolimits_{L}\left({f}_{d,6} \left({r}_{ij,L}\right)\frac{{C}_{6ij}}{{r}_{ij,L}^{6}}+ {f}_{d,8} \left({r}_{ij,L}\right)\frac{{C}_{8ij}}{{r}_{ij,L}^{8}}\right)$$

The dispersion coefficients ${C}_{6ij}$ are geometry dependent as they are adjusted based on the local geometry (coordination number) around atoms* i* and *j*.

A type of scalar fields called ELF may demonstrate a broad span of bonding samples [35, 36]. "Becke and Edgecombe" remarked that spherically averaged like spin conditional pair probability possesses a direct correlation with the Fermi hole and proposed the parameter of ELF in Multiwfn program [37, 38] and popularized for spin-polarized procedure [39, 40]:

2 $$\text{ELF}\left(\text{r}\right)=\frac{1}{1+\left[D(\text{r})/{D}_{0}(\text{r})\right]}$$

where

3 $$D\left(\text{r}\right)=\frac{1}{2} \sum\nolimits_{i}{\eta }_{i}{\left|\nabla {\varphi }_{i} (\text{r})\right|}^{2}- \frac{1}{8}\left[\frac{{\left|\nabla {\rho }_{\alpha } (\text{r})\right|}^{2}}{{\rho }_{\alpha } (\text{r})}+ \frac{{\left|\nabla {\rho }_{\beta } (\text{r})\right|}^{2}}{{\rho }_{\beta } (\text{r})}\right],$$

and

4 $${D}_{0}\left(\text{r}\right)=\frac{3}{10}{\left(6{\pi }^{2}\right)}^{2/3}\left[{{\rho }_{\alpha } (\text{r})}^{5/3}+ {{\rho }_{\beta } (\text{r})}^{5/3}\right].$$

For close-shell system, since ${\rho }_{\alpha }={\rho }_{\beta }=\left(1/2\right)\rho$, *D* and *D*_0_ terms can be simplified as:

5 $$D\left(\text{r}\right)=\frac{1}{2} \sum_{i}{\eta }_{i}{\left|\nabla {\varphi }_{i} (\text{r})\right|}^{2}- \frac{1}{8} \frac{{\left|\nabla \rho \left(\text{r}\right)\right|}^{2}}{\rho \left(\text{r}\right)},$$

and

6 $${D}_{0}\left(\text{r}\right)=\left(3/10\right){\left(3{\pi }^{2}\right)}^{2/3 }{\rho \left(\text{r}\right)}^{5/3}.$$

In an isolated system (such as molecule), the energy levels are discrete, the concept of density of state (DOS) is supposed to be completely valueless in this situation. Therefore, the original total DOS (TDOS) of isolated system can be written as [41]:

7 $$TDOS \left(E\right)= \sum_{i}\delta (E-{\epsilon }_{i })$$

The normalized Gaussian function is defined as:

$$G\left(x\right)=\frac{1}{c\sqrt{2\pi }}{e}^{-\frac{{x}^{2}}{2{c}^{2}}}$$

where

8 $$c=\frac{\text{FWHM}}{2\sqrt{2\text{lnx}}}$$

FWHM (full width at half maximum) is an adjustable parameter in Multiwfn [37, 38]. Moreover, the curve map of broadened partial DOS (PDOS) and overlap DOS (OPDOS) are valuable for visualizing orbital composition analysis, PDOS function of fragment *A* is defined as:

9 $${PDOS}_{A} \left(E\right)=\sum_{i}{\Xi }_{i,A} F (E-{\epsilon }_{i })$$

where ${\Xi }_{i,A}$ is the composition of fragment *A* in orbital *i*. The OPDOS between fragment *A* and *B* is defined as:

10 $${OPDOS}_{A,B} \left(E\right)=\sum_{i}{\text{X}}_{A,B}^{i} F (E-{\epsilon }_{i })$$

where ${\text{X}}_{A,B}^{i}$ is the composition of total cross term between fragment *A* and *B* in orbital *i*.

**Fig. 1:**
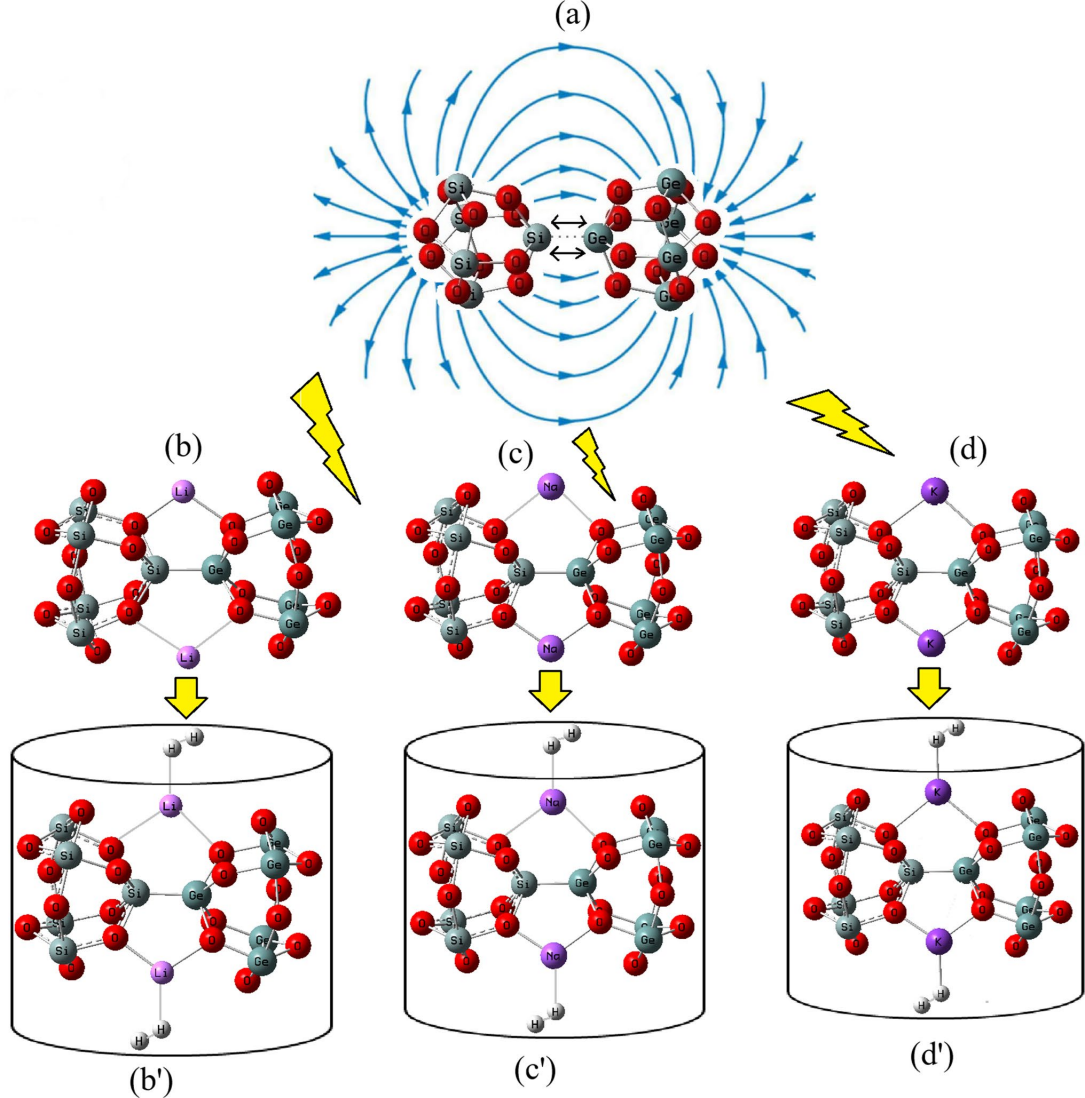
Adding Li, Na, K to (**a**) [GeO–SiO] nanocluster and formation of (**b**) Li[GeO–SiO], (**c**) Na[GeO–SiO], (**d**) K[GeO–SiO] towards hydrogen adsorption and energy storage in novel batteries of (b′) Li[GeO–SiO]–2H_2_, (c′) Na[GeO–SiO]–2H_2_, and (d′) K[GeO–SiO]–2H_2_

The aim of this study is to hydrogen adsorption by using alkali metals-based nanoclusters of Li[GeO–SiO], Na[GeO–SiO] or K[GeO–SiO] (Fig. 1) which can increase the hydrogen storage in cell batteries, transistors or other semiconductors. In our research, the calculations have been done by CAM–B3LYP–D3 /EPR–3 level of theory. Figure 1 shows the process of hydrogen adsorption by Li[GeO–SiO], Na[GeO–SiO] or K[GeO–SiO] nanocluster and hydrogen-adsorbed nanoclusters of Li[GeO–SiO]–2H_2_, Na[GeO–SiO]–2H_2_ or K[GeO–SiO]–2H_2_. The Bader charge analysis [42] was discussed during trapping of hydrogen atoms by Li[GeO–SiO], Na[GeO–SiO] or K[GeO–SiO] nanoclusters (Fig. 1). The rigid potential energy surface using density functional theory [43–45] was performed due to Gaussian 16 revision C.01 program package [46] and GaussView 6.1 [47]. The coordination input for hydrogen grabbing by Li[GeO–SiO], Na[GeO–SiO], and K[GeO–SiO] has lanl2dz and applied 6–311 + G (d,p) basis sets.

## Results and Discussion

### CDD Analysis

**Fig. 2:**
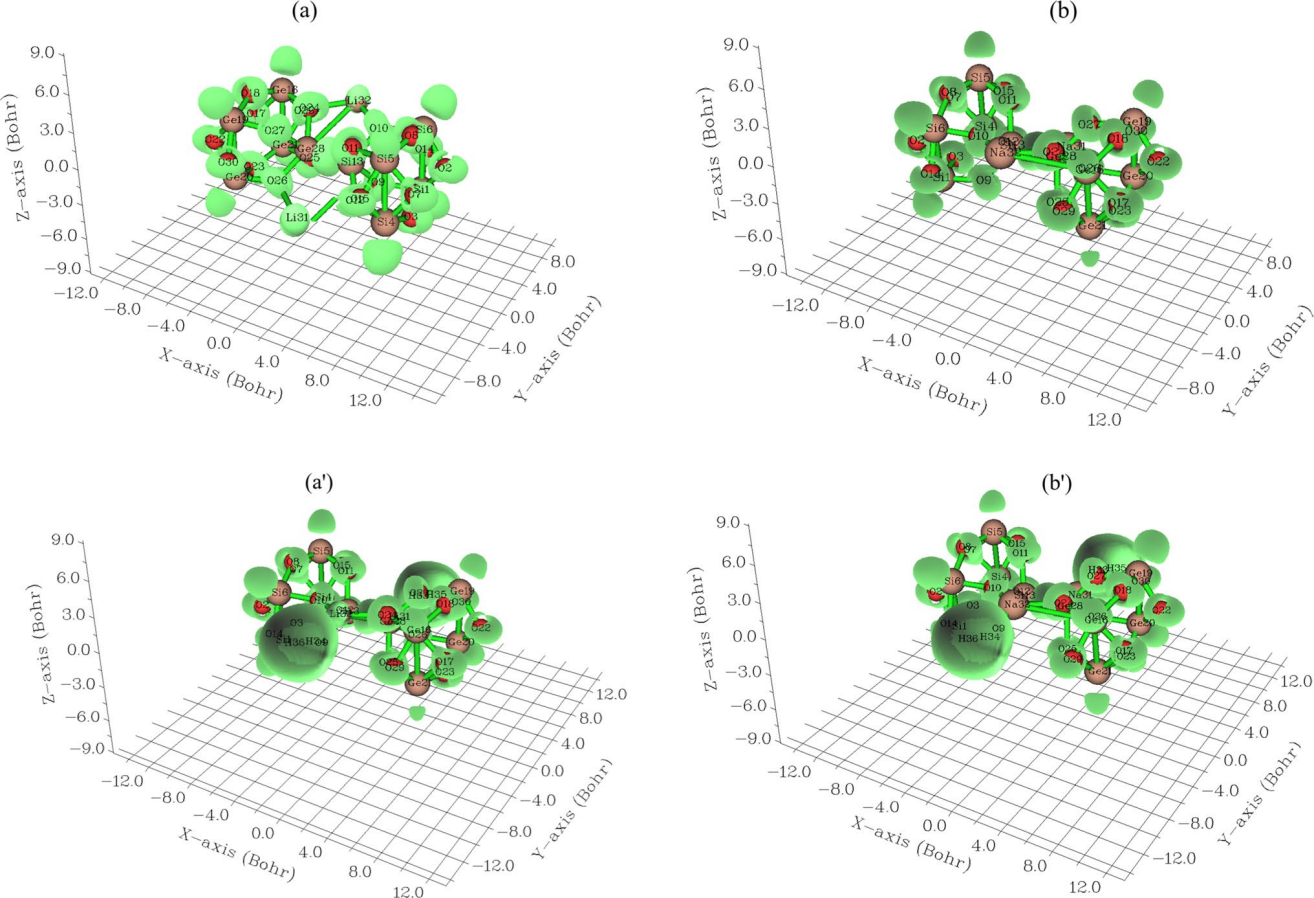

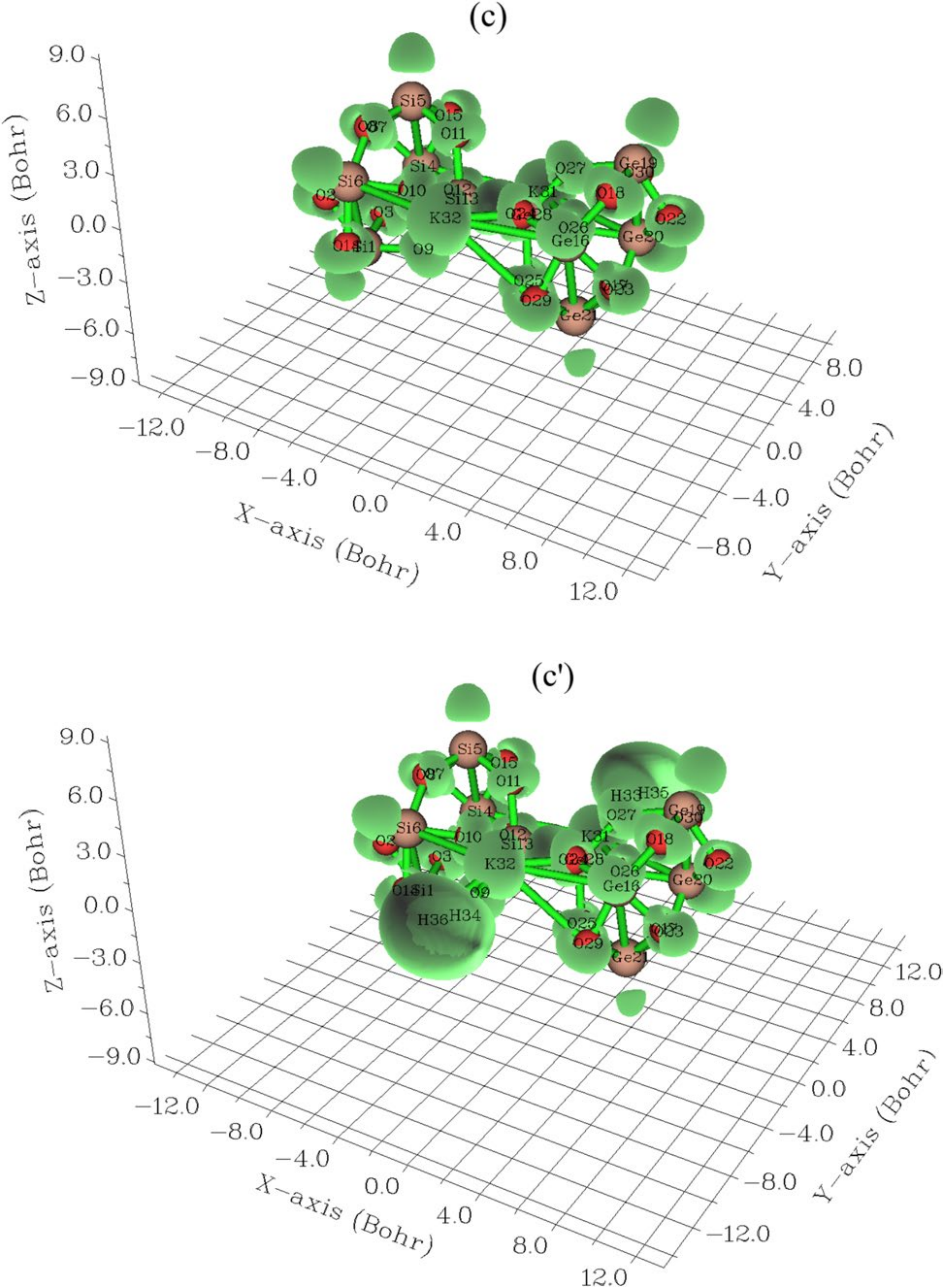
CDD graphs for (**a**) Li[GeO–SiO], (**a**′) Li[GeO–SiO]–2H_2_, (**b**) Na[GeO–SiO], (**b**′) Na[GeO–SiO]–2H_2_, (**c**) K[GeO–SiO], and (**c**′) K[GeO–SiO]–2H_2_ nanoclusters

In Fig. 2a, Li[GeO–SiO] cluster with the fluctuation in the region around –12 to + 6 Bohr forms the nanocluster of Li[GeO–SiO]–2H_2_ (Fig. 2a′) in the area around –12 to + 3 Bohr. Furthermore, the atoms of O2, O3, O7–O12, O14, O15, O17, O18, O22–O27, O29, O30 from Na[GeO–SiO] (Fig. 2b) have shown the fluctuation around –12 to + 4 Bohr towards formation of Na[GeO–SiO]–2H_2_ through hydrogen adsorption (Fig. 2b′). In addition, K[GeO–SiO] cluster with the fluctuation in the region around –12 to + 4 Bohr (Fig. 2c) forms the nanocluster of K[GeO–SiO]–2H_2_ during hydrogen grabbing (Fig. 2c′) in the same range of area around –12 to + 4 Bohr. Atomic charge was discussed during trapping of hydrogens by Li[GeO–SiO], Na[GeO–SiO] or K[GeO–SiO] nanoclusters towards formation of Li[GeO–SiO]–2H_2_, Na[GeO–SiO]–2H_2_ or K[GeO–SiO]–2H_2_, respectively (Table 1) [48]. This shows that almost all of the charge acquired by the H atoms come from the surface and subsurface atoms. The region of charge density variation is localized mainly between the H atoms and the first and second layers of Li[GeO–SiO], Na[GeO–SiO]or K[GeO–SiO] atoms.

**Table 1:** The atomic charge (Q/coulomb) for The atomic charge (Q/coulomb) for Li[GeO–SiO], Li[GeO–SiO]–2H_2_, Na[GeO–SiO], Na[GeO–SiO]–2H_2_, K[GeO–SiO]nanoclusters, and K[GeO–SiO]–2H_2_ nanoclusters

Li[ GeO–SiO]		Li[GeO–SiO]–2H_2_		Na[GeO–SiO]		Na[GeO–SiO]–2H_2_		K[GeO–SiO]		K[GeO–SiO]–2H_2_	
Atom	Charge	Atom	Charge	Atom	Charge	Atom	Charge	Atom	Charge	Atom	Charge
Si1	1.4586	Si1	1.4565	Si1	1.4601	Si1	1.4585	Si1	1.4710	Si1	1.4709
O2	–0.6856	O2	–0.6559	O2	–0.6561	O2	–0.6875	O2	–0.6289	O2	–0.6290
O3	–0.8317	O3	–0.8358	O3	–0.8359	O3	–0.8320	O3	–0.8318	O3	–0.8319
Si4	1.4365	Si4	1.4303	Si4	1.4184	Si4	1.4282	Si4	1.4174	Si4	1.4184
Si5	1.4612	Si5	1.4458	Si5	1.4531	Si5	1.4656	Si5	1.4487	Si5	1.4481
Si6	1.4613	Si6	1.4638	Si6	1.4554	Si6	1.4561	Si6	1.4700	Si6	1.4709
O7	–0.6525	O7	–0.6830	O7	–0.6815	O7	–0.6534	O7	–0.7146	O7	–0.7147
O8	–0.8377	O8	–0.8434	O8	–0.8442	O8	–0.8385	O8	–0.8347	O8	–0.8348
O9	–0.7899	O9	–0.7870	O9	–0.7889	O9	–0.7911	O9	–0.7864	O9	–0.7864
O10	–1.0109	O10	–1.0019	O10	–0.9953	O10	–0.9970	O10	–1.0538	O10	–1.0543
O11	–0.8020	O11	–0.8024	O11	–0.8070	O11	–0.8067	O11	–0.8086	O11	–0.8085
O12	–0.9454	O12	–0.9524	O12	–0.9671	O12	–0.9570	O12	–0.9919	O12	–0.9916
Si13	1.6312	Si13	1.6329	Si13	1.6261	Si13	1.6351	Si13	1.5744	Si13	1.5739
O14	–0.7005	O14	–0.7301	O14	–0.7318	O14	–0.7084	O14	–0.7651	O14	–0.7672
O15	–0.7611	O15	–0.7232	O15	–0.7297	O15	–0.7669	O15	–0.7011	O15	–0.7018
Ge16	1.3977	Ge16	1.4175	Ge16	1.4172	Ge16	1.4025	Ge16	1.4046	Ge16	1.4053
O17	–0.6718	O17	–0.6526	O17	–0.6530	O17	–0.6715	O17	–0.6077	O17	–0.6075
O18	–0.7819	O18	–0.7795	O18	–0.7796	O18	–0.7837	O18	–0.7826	O18	–0.7824
Ge19	1.3823	Ge19	1.3975	Ge19	1.4017	Ge19	1.3825	Ge19	1.3823	Ge19	1.3825
Ge20	1.3900	Ge20	1.3931	Ge20	1.3876	Ge20	1.3859	Ge20	1.3623	Ge20	1.3650
Ge21	1.3916	Ge21	1.4017	Ge21	1.4035	Ge21	1.3883	Ge21	1.4024	Ge21	1.4002
O22	–0.6229	O22	–0.6688	O22	–0.667	O22	–0.6235	O22	–0.6959	O22	–0.6964
O23	–0.7867	O23	–0.7833	O23	–0.7827	O23	–0.7863	O23	–0.7867	O23	–0.7866
O24	–0.9474	O24	–0.9440	O24	–0.9548	O24	–0.9516	O24	–0.9944	O24	–0.9944
O25	–0.7899	O25	–0.7749	O25	–0.7797	O25	–0.7929	O25	–0.7850	O25	–0.7848
O26	–0.9187	O26	–0.9064	O26	–0.9315	O26	–0.9381	O26	–0.9694	O26	–0.9693
O27	–0.7748	O27	–0.7935	O27	–0.7889	O27	–0.7704	O27	–0.7819	O27	–0.7823
Ge28	1.2474	Ge28	1.2558	Ge28	1.2887	Ge28	1.2880	Ge28	1.2213	Ge28	1.2212
O29	–0.6903	O29	–0.7345	O29	–0.7405	O29	–0.6994	O29	–0.7578	O29	–0.7603
O30	–0.7318	O30	–0.7221	O30	–0.7249	O30	–0.7380	O30	–0.6764	O30	–0.6782
Li31	0.7345	Li31	0.6437	Na31	0.8096	Na31	0.7180	K31	0.9096	K31	0.8790
Li32	0.7418	Li32	0.6386	Na32	0.7187	Na32	0.6409	K32	0.8911	K32	0.8709
		H33	–0.0264			H33	–0.0277			H33	–0.0609
		H34	–0.0074			H34	–0.0087			H34	–0.0399
		H35	0.1277			H35	0.1000			H35	0.0903
		H36	0.1036			H36	0.0818			H36	0.0666

**Fig. 3:**
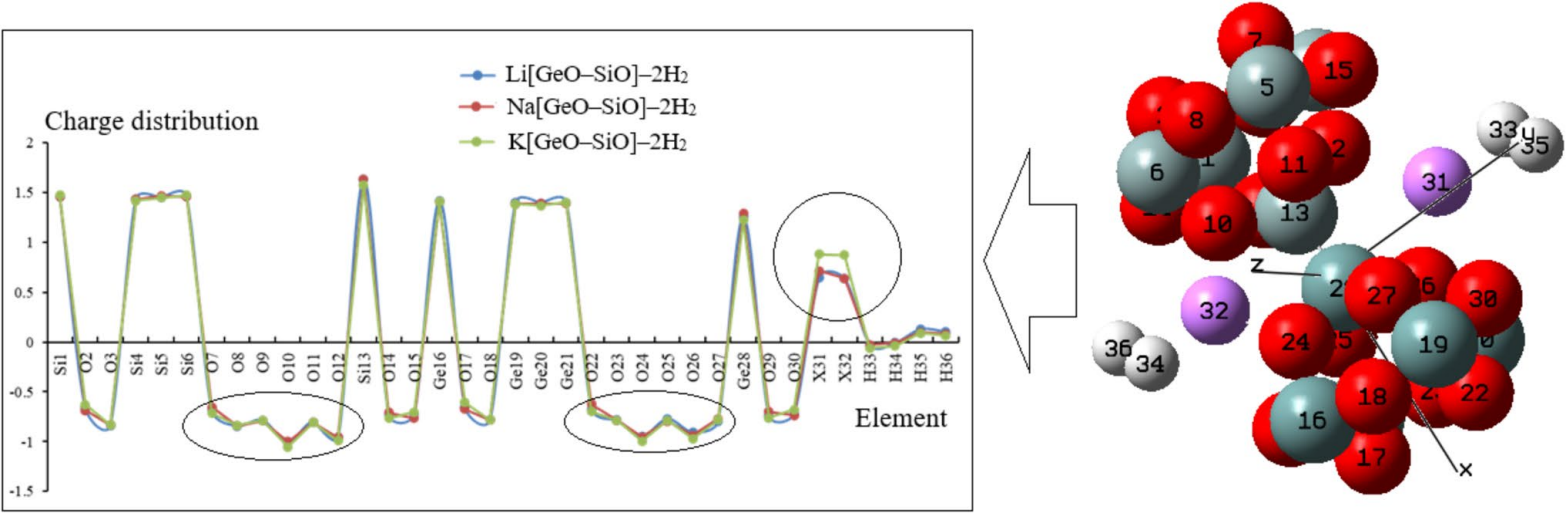
The fluctuation of atomic charge (Q/coulomb) for Li[GeO–SiO], Na[GeO–SiO] or K[GeO–SiO] nanoclusters. (Note: X = Li, Na, K)

The atomic charge of Si, Ge, O, and alkali metals of Li, Na, K and hydrogen atoms absorbed on Li[GeO–SiO], Na[GeO–SiO] or K[GeO–SiO] nanoclusters have been measures. The values detect that with adding lithium, sodium and potassium, the negative atomic charge of oxygen atoms of O2, O3, O7–O12, O14, O15, O17, O18, O22–O27, O29, O30 in Li[GeO–SiO]–2H_2_, Na[GeO–SiO]–2H_2_ or K[GeO–SiO]–2H_2_ nanoclusters augments. In fact, Li[GeO–SiO], Na[GeO–SiO] or K[GeO–SiO] nanoclusters have shown more efficiency than GeO–SiO cluster [30] for admitting the electron from electron donor of H33, H34, H35 and H36 (Table 1 and Fig. 3).

The changes of charge density analysis in the adsorption process have illustrated that Li[GeO–SiO] has shown the "Bader charge" of –1.631 C before hydrogen adsorption and –1.633 C after hydrogen adsorption. Moreover, the changes of charge density analysis for Na[GeO–SiO] has shown the "Bader charge" of –1.626 C before hydrogen adsorption and –1.635 C after hydrogen adsorption. However, K[GeO–SiO] has shown the "Bader charge" of –1.574 C before hydrogen adsorption and after hydrogen adsorption. The differences of charge density for these structures are measured as: ΔQ _Li [GeO–SiO]_ = –0.002, ΔQ _Na [GeO–SiO]_ = –0.009 and ΔQ _K [GeO–SiO]_ = –0.00. Therefore, the results have shown that the cluster of Na[GeO–SiO] and Li[GeO–SiO] may have the most tensity for electron accepting owing to hydrogen grabbing.

### TDOS/OPDOS Analysis

**Fig. 4:**
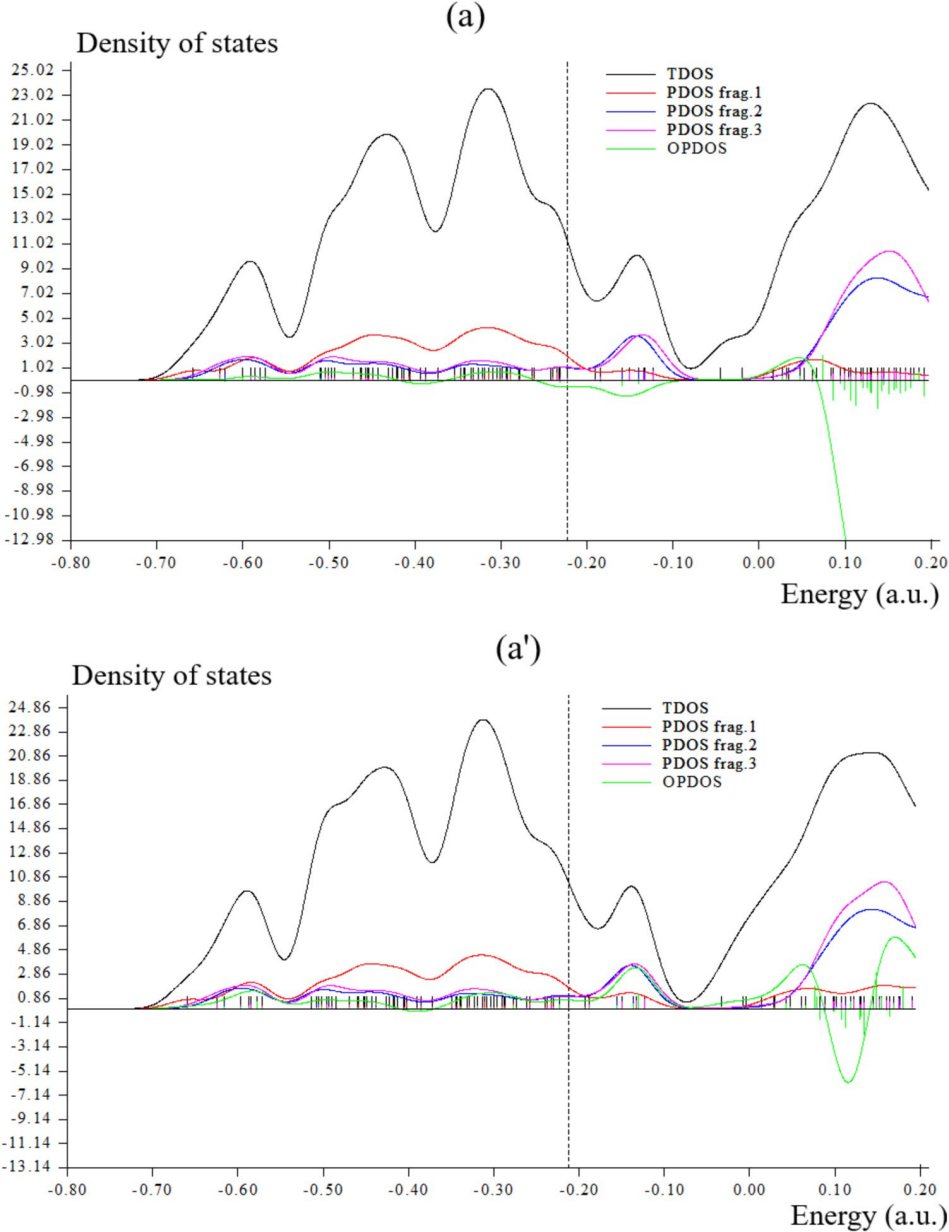

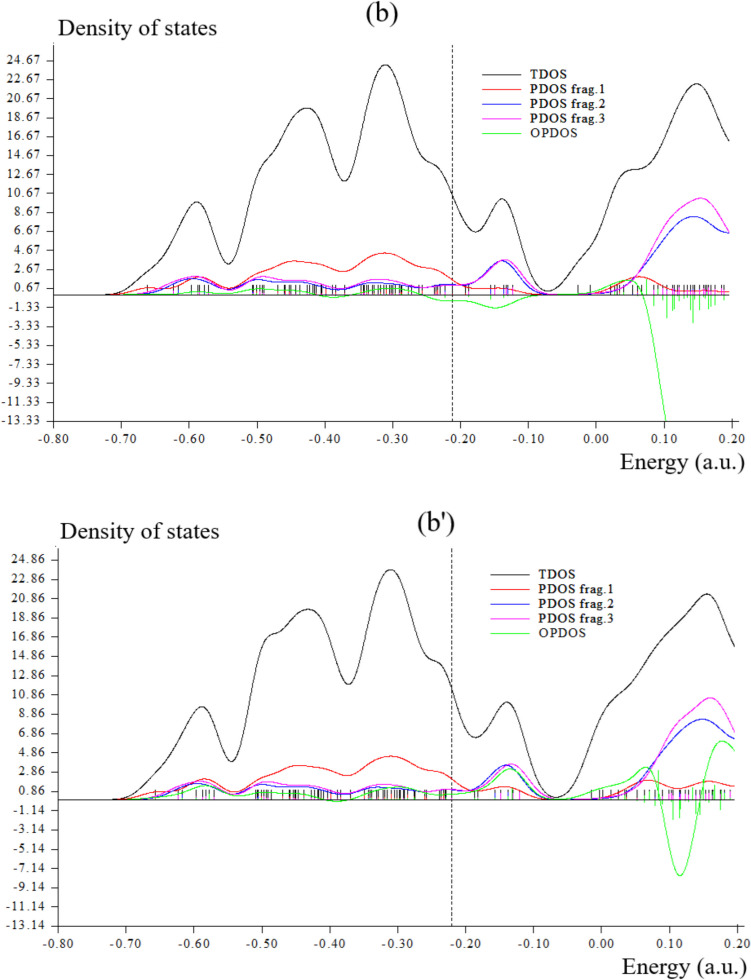

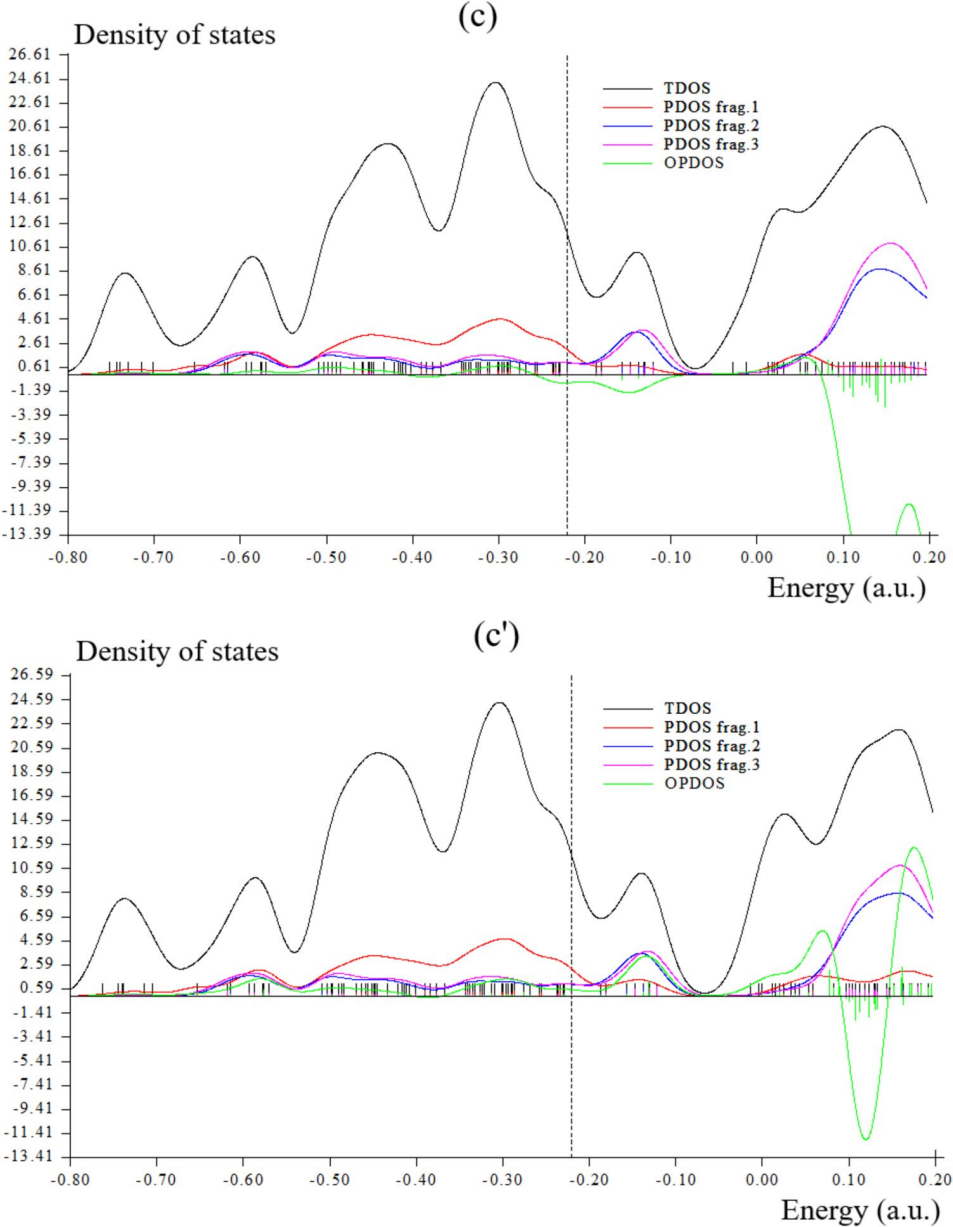
TDOS graphs of (**a**) Li[GeO–SiO], (**a**′) Li[GeO–SiO]–2H_2_, (**b**) Na[GeO–SiO], (**b**′) Na[GeO–SiO]–2H_2_, (**c**) K[GeO–SiO], and (**c**′) K[GeO–SiO]–2H_2_ nanoclusters

During an adsorption study, it was revealed that there was a notable interaction between the pure graphene nanostructure and Br_2_ gas, while the S-doped counterpart exhibited reduced interaction [49–51]. In this work, during formation of Li[GeO–SiO] cluster, Fig. 4a has shown sharp and sophisticated peaks around –0.3, –0.45 and –0.60 a.u. due to covalent bond between two atoms of Li with GeO–SiO cluster. Moreover, Li[GeO–SiO]–2H_2_ has indicated a duplicate peak around –0.45 to –0.5 a.u. during hydrogen adsorption (Fig. 4a′). After H-grabbing by Na[GeO–SiO] cluster, pointed peaks around –0.3, –0.45 and –0.60 a.u. due to covalent bond between two atoms of Na with GeO–SiO cluster (Fig. 4b). Furthermore, Na[GeO–SiO]–2H_2_ has indicated a duplicate peak around –0.45 to –0.5 a.u. during hydrogen adsorption (Fig. 4b′).However,

The maximum energy of TDOS for K[GeO–SiO] (Fig. 4c) with several peaks around –0.35, –0.45, –0.6 and –0.75 a.u. with maximum density of state of ≈ 23 around –0.35 a.u. has been shown. Moreover, similar amounts of TDOS for K[GeO–SiO]–2H_2_ (Fig. 4c′) through some fluctuations in the behavior of the graphs have been observed.

Fragment 1 has been defined for O9 to O13, O24 to O27 and Ge28, X31 and X32 (X = Li, Na, K) in Fig. 4a, b, c and H36 to H36 in Fig. 4a′, b′, c′. Fragment 2 has indicated the fluctuation of Si1, Si4 to Si6 beside the similar involved atoms of Fragment 1 in Fig. 4a, b, c and Fig. 4a′, b′, c′. Finally, it was considered the fluctuation of Ge16, Ge19 to Ge21, O17, O18, O22, O23, O29, O30 in Fig. 4a, a′, b, b′, c, c′ through Fragment 3. The PDOS of the H_2_ molecule adsorption system shows that when H_2_ molecule is close to the surface, the 1 s orbital electrons of H_2_ molecule will move to a lower energy level and hybridize with the 5f/6d orbital electrons of U atoms in the nearby surface and subsurface layers, forming a new hybridized orbital peak, which is formed near − 0.16 a.u. for H_2_ molecule dissociative adsorption.

### ELF Analysis

**Fig. 5:**
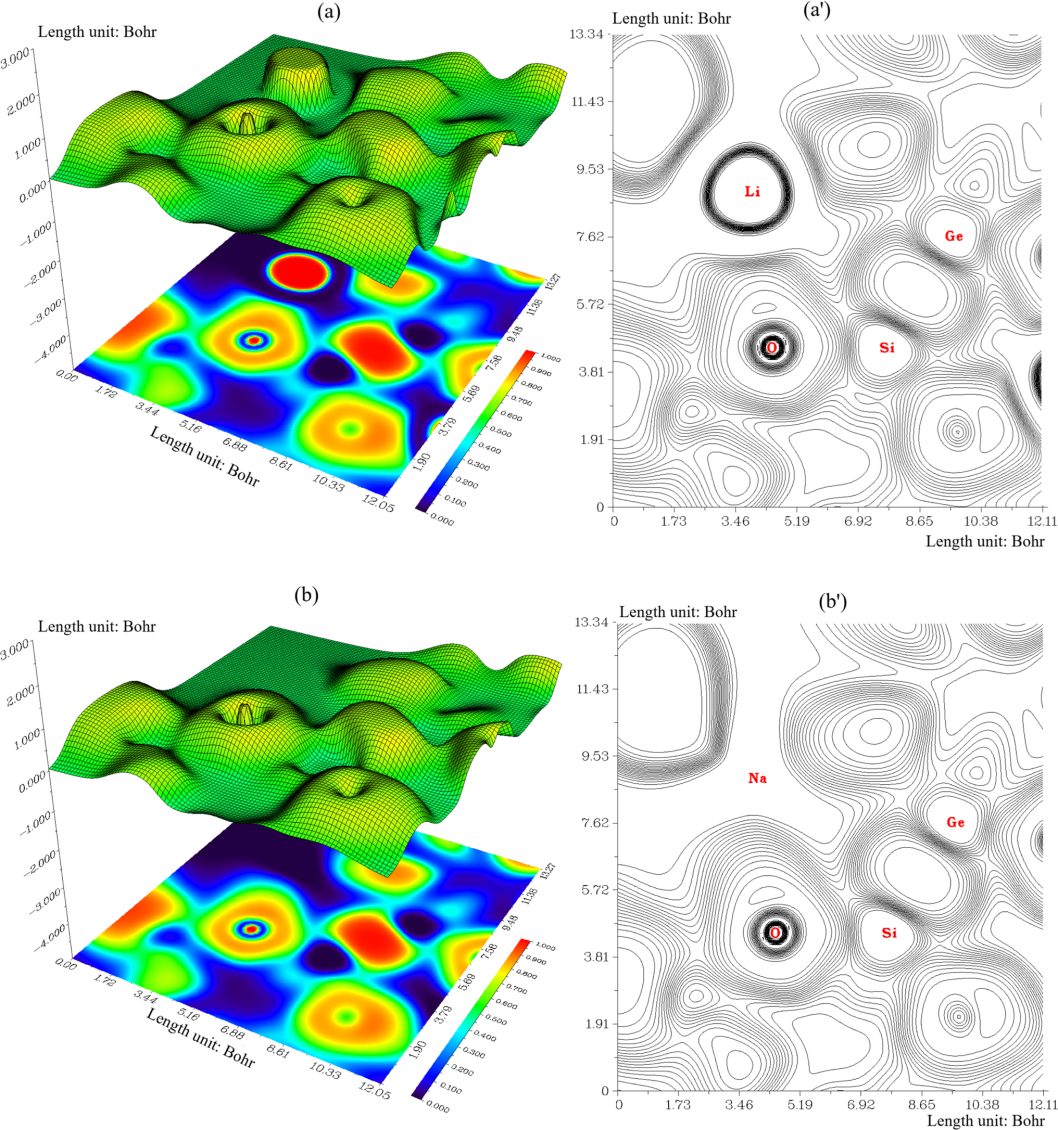

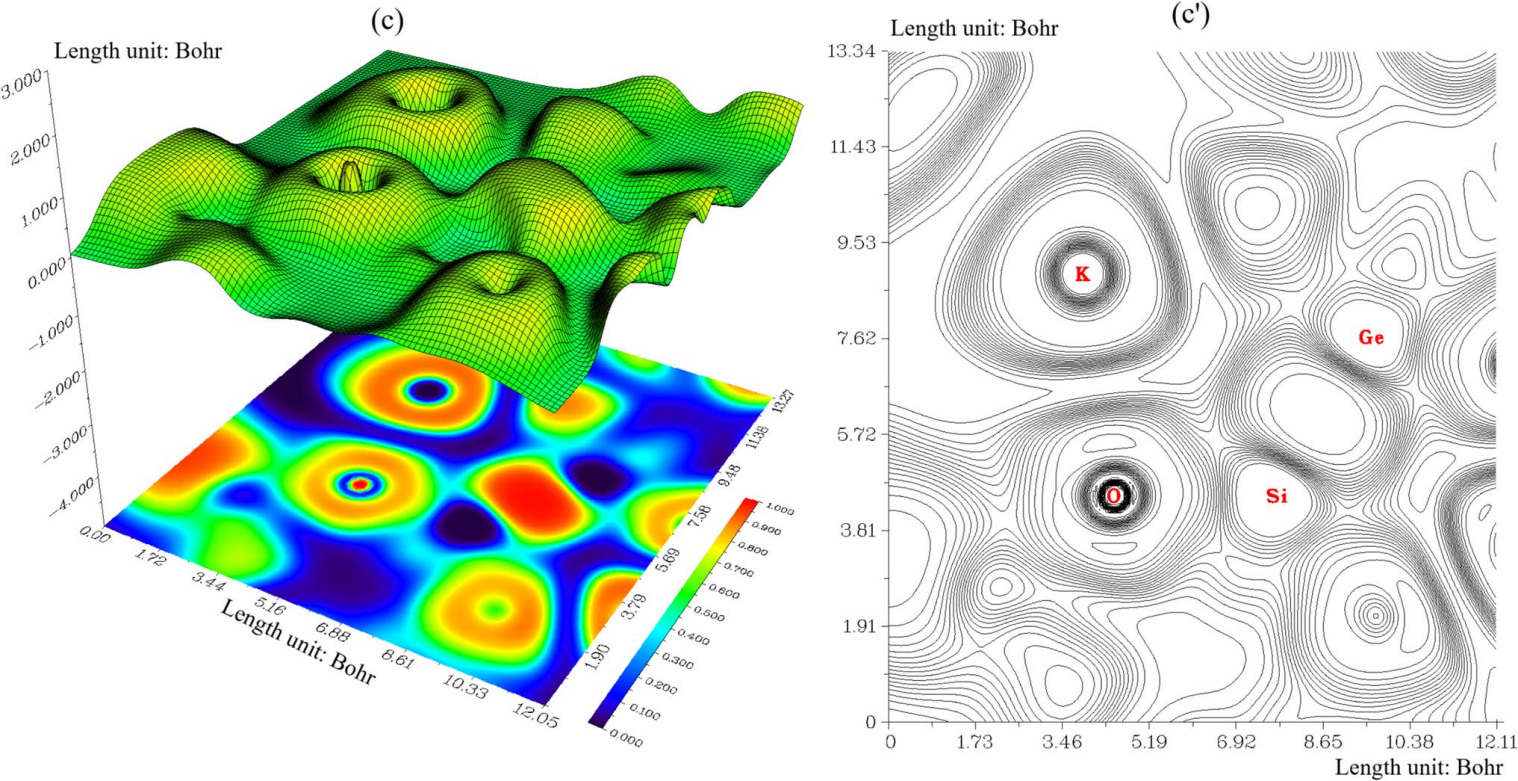
The graphs of ELF for (**a**) Li[GeO–SiO], (**a**′) Li[GeO–SiO]–2H_2_, (**b**) Na[GeO–SiO], (**b**′) Na[GeO–SiO]–2H_2_, (**c**) K[GeO–SiO], and (**c**′) K[GeO–SiO]–2H_2_ nanoclusters. (Counter line map in the right and shaded surface map with projection in the left)

Trapping of hydrogens by Li[GeO–SiO], Na[GeO–SiO] or K[GeO–SiO] nanoclusters towards formation of Li[GeO–SiO]–2H_2_, Na[GeO–SiO]–2H_2_ or K[GeO–SiO]–2H_2_ can be defined by ELF graphs owing to exploring their delocalization/localization characterizations of electrons and chemical bonds (Fig. 5a, a′, b, b′, c, c′).

A vaster jointed area engaged by an isosurface map has shown the electron delocalization in Li[GeO–SiO] (Fig. 5a), Li[GeO–SiO] –2H_2_ (Fig. 5a′), Na[GeO–SiO] (Fig. 5b), Na[GeO–SiO] –2H_2_ (Fig. 5b′), K[GeO–SiO] (Fig. 5c), and K[GeO–SiO] –2H_2_ (Fig. 5c′) through labeling atoms of O12, Si13, O26, Ge28, X31(X = Li, Na or K) and H35. In fact, the counter map of ELF can confirm that Li[GeO–SiO], Na[GeO–SiO], or K[GeO–SiO] nanocluster may increase the efficiency during hydrogen adsorption towards formation of Li[GeO–SiO]–2H_2_, Na[GeO–SiO]–2H_2_, or K[GeO–SiO]–2H_2_ nanocluster.

The applied wavefunction level is CAM–B3LYP–D3/6–311 + G (d, p) that corresponds to HOMO and LUMO, respectively (Table 2). Energy band gaps were found in spin up direction at the Fermi level which reveals the half-metallicity of Li/Na/K[GeO–SiO] nanoclusters that were found stable in the ferromagnetic phase as compared to the non-magnetic phase. Photocatalytic hydrogen adsorption might be a promising eco-friendly approach for renewable energy production in batteries [52]. Based on the results in Table 2, K[GeO–SiO]–2H_2_ and Na[GeO–SiO]–2H_2_ heterostructures with band gap of 0.9230 and 0.8963 eV, respectively can be more efficient for saving energy.

**Table 2:** LUMO, HOMO, energy gap (∆E), Ring perimeter (Å), Total ring area (Å^2^) for Li[GeO–SiO], Na[GeO–SiO], K[GeO–SiO] through hydrogen grabbing and formation of Li[GeO–SiO]–2H_2_, Na[GeO–SiO]–2H_2_, K[GeO–SiO]–2H_2_ heteroclusters

Heteroclusters	Ring perimeter (Å)	Total ring area (Å^2^)	E_HOMO_ (eV)	E_LUMO_ (eV)	∆E = E_LUMO_ –E_HOMO_ (eV)
Li[GeO–SiO]	9.3332	5.1566	–6.0781	–5.1750	0.9031
Li[GeO–SiO]–2H_2_	9.9121	6.6752	–5.7793	–5.2402	0.5390
Na[GeO–SiO]	9.9122	6.6754	–5.7812	–5.2378	0.5434
Na[GeO–SiO]–2H_2_	9.9121	6.6752	–5.9990	–5.1021	0.8963
K[GeO–SiO]	9.9122	6.6754	–6.0005	–5.0738	0.9267
K[GeO–SiO]–2H_2_	9.9121	6.6752	–5.9990	–5.0760	0.9230

The amount of "Mayer bond order" [53] is generally according to empirical bond order for the single bond is near 1.0. "Mulliken bond order" [54] with a small accord with empirical bond order is not appropriate for quantifying bonding strength, for which Mayer bond order always performs better. However, "Mulliken bond order" is a good qualitative indicator for "positive amount" of bonding and "negative amount" of antibonding which are evacuated and localized, respectively (Tables 3 and 4).

**Table 3:** The bond order of Mayer, Wiberg, Mulliken, Laplacian and Fuzzy from mixed alpha and beta density matrix for Li[GeO–SiO], Na[GeO–SiO], K[GeO–SiO] heterostructures

Compound	Bond type	Bond order				
		Mulliken	Fuzzy	Wiberg	Laplacian	Mayer
Li[GeO–SiO]	O12–Si13	0.1665	0.9710	0.6182	0.2221	0.4808
	O12–Li31	0.1313	0.1312	0.2026	0.2376	0.1394
	O26–Ge28	0.2292	1.0523	0.5818	0.2465	0.4768
	O26–Li31	0.1890	0.1805	0.2763	0.1645	0.2166
Na[GeO–SiO]	O12–Si13	0.1856	0.9749	0.6129	0.1967	0.4901
	O12–Na31	0.1078	0.3085	0.1560	0.1402	0.1016
	O26–Ge28	0.2348	0.9938	0.5751	0.2198	0.4635
	O26–Na31	0.1549	0.3832	0.2016	0.1438	0.1618
K[GeO–SiO]	O12–Si13	0.1963	0.9632	0.611	0.1849	0.4989
	O12–K31	0.0730	0.3697	0.1403	0.1863	0.2375
	O26–Ge28	0.2610	0.9878	0.5784	0.2038	0.4990
	O26–K31	0.1444	0.4507	0.1846	0.1391	0.1057

**Table 4:** The bond order of Mayer, Wiberg, Mulliken, Laplacian and Fuzzy from mixed alpha and beta density matrix for hydrogenated of Li[GeO–SiO]–2H_2_, Na[GeO–SiO]–2H_2_, K[GeO–SiO]–2H_2_ heterostructures

Compound	Bond type	Bond order				
		Mulliken	Fuzzy	Wiberg	Laplacian	Mayer
Li[GeO–SiO]–2H_2_	O12–Si13	0.1564	1.0140	0.6110	0.2119	0.4766
	O12–Li31	0.1390	0.1328	0.2034	0.1956	0.1439
	O26–Ge28	0.2138	1.0421	0.5722	0.2404	0.4616
	O26–Li31	0.1910	0.1772	0.2699	0.2985	0.2138
	Li31–H33	0.0984	0.2085	0.1796	0.0856	0.1028
Na[GeO–SiO]–2H_2_	O12–Si13	0.1818	0.9791	0.6181	0.2073	0.4857
	O12–Na31	0.1114	0.3042	0.1558	0.1376	0.1064
	O26–Ge28	0.2399	1.0033	0.5841	0.2223	0.4723
	O26–Na31	0.1656	0.3820	0.2029	0.1640	0.1723
	Na31–H33	0.0705	0.2048	0.1613	0.0648	0.0749
K[GeO–SiO]–2H_2_	O12–Si13	0.1906	0.9630	0.6107	0.1848	0.4963
	O12–K31	0.0673	0.3683	0.1393	0.1045	0.1684
	O26–Ge28	0.2578	0.9875	0.5780	0.2040	0.4970
	O26–K31	0.1390	0.4489	0.1821	0.1024	0.1092
	K31–H33	0.0538	0.2042	0.1706	0.0429	0.0557

As it is seen in Tables 3 and 4, “Laplacian bond order” [55] has a straight cohesion with bond polarity, bond dissociation energy and bond vibrational frequency. The low value of Laplacian bond order might demonstrate that it is insensitive to the calculation degree applied for producing electron density. Generally, the value of “Fuzzy bond order” is near Mayer bond order, especially for low-polar bonds, but much more stable with respect to the change in basis-set. Computation of “Fuzzy bond order” demands running “Becke's DFT” numerical integration, owing to which the calculation value is larger than assessment of “Mayer bond order” and it can concede more precisely [56].

### Thermodynamic Properties

Table 5 through the thermodynamic specifications concluded that [GeO–SiO]-based heterostructures might be a more efficient structure for hydrogen trapping. Thermodynamic parameters of hydrogen adsorption on Li[GeO–SiO], Na[GeO–SiO] and K[GeO–SiO] heterostructures have been assigned through a given number of hydrogen donor sites and formation of Li[GeO–SiO]–2H_2_, Na[GeO–SiO]–2H_2_ and K[GeO–SiO]–2H_2_ heterostructures (Table 5).

**Table 5:** Thermodynamic properties (kcal/mol) of Li[GeO–SiO], Na[GeO–SiO], K[GeO–SiO] through hydrogen grabbing and formation of Li[GeO–SiO]–2H_2_, Na[GeO–SiO]–2H_2_, K[GeO–SiO]–2H_2_ heteroclusters

Heterostructure	∆E^o^_ads_ × 10^–3^	E^o^_H–binding_ × 10^–3^	∆H^o^_ads_ × 10^–3^	∆G^o^_ads_ × 10^–3^
Li[GeO–SiO]	–1379.64	–0.29 × 10^+3^	–1379.64	–1379.64
Li[GeO–SiO]–2H_2_	–1379.93	-	–1379.93	–1379.96
Na[GeO–SiO]	–6981.16	–0.27 × 10^+3^	–6981.16	–6981.20
Na[GeO–SiO]–2H_2_	–6981.44	-	–6981.44	–6981.47
K[GeO–SiO]	–8360.86	–0.50 × 10^+3^	–8360.86	–8360.89
K[GeO–SiO]–2H_2_	–8361.37	-	–8361.37	–8361.39

The changes of Gibbs free energy could detect the maximum efficiency of GeO–SiO]-based heterostructures for hydrogen adsorption through ${\Delta \text{G}}_{\text{ads}}^{\text{o}}$ which is related to linkage between hydrogen atoms with silicon and germanium in GeO–SiO]-based heterostructures and formation of hydrogenated compounds of Li[GeO–SiO]–2H_2_, Na[GeO–SiO] –2H_2_ and K[GeO–SiO] –2H_2_. Although at atomic levels, all ionic and molecular interactions can be interpreted as electric, this term is restricted to coulombic interactions and all other interactions are termed non-electrostatic, whatever their origin. These interactions, either attractive or repulsive, are strongly dependent on the charge densities for both the GeO–SiO]-based heterostructures surface and the adsorptive molecule of hydrogen. The non-electrostatic interactions are always attractive, and include van der Waals forces, hydrophobic interactions and hydrogen bonding (Table 5).

Based on the values of hydrogen binding (E^o^_H–binding_) in Table 5 (–0.5 × 10^+3^, –0.27 × 10^+3^ and –0.29 × 10^+3^ kcal/mol), the stability of hydrogenated heterostructures of Si_5_O_10_, Ge_5_O_10_ and Si_5_O_10_–Ge_5_O_10_ nanoclusters can be considered as K[GeO–SiO]–2H_2_ > Na[GeO–SiO] –2H_2_ > Li[GeO–SiO] –2H_2_.

Table 5 has shown the key role of interaction between the adsorbate of hydrogen atoms as the electron donors and the adsorbent of Li[GeO–SiO], Na[GeO–SiO] and K[GeO–SiO] heterostructures as the electron acceptors. Moreover, germanium also has interesting optical properties, including transparency to infrared radiation. This characteristic makes it valuable in the production of lenses for thermal imaging systems and night vision devices. Its use extends to fiber optics, where it serves as a dopant to enhance the refractive index of optical fibers, improving signal transmission. Metal oxides have shown great potential as coating candidates due to their high electric conductivity, ability to enhance structural stability and good electrochemical performance when compared to the majority of other surface modifications. So, various aspects in relation to performance enhancement effects of different metal oxides that are used as coatings on materials for hydrogen adsorption properties are essential. Hence, formation of composites by combining two or more compatible materials in the hopes of enhancing thermodynamic properties of functional materials for energy storage and superior performance is remarkable. Finally, anchoring Li/Na/Na on [GeO–SiO] nanocluster can promise enhancing hydrogen storage in the cell batteries through formation of the hydrogenated clusters of Li[GeO–SiO]–2H_2_, Na[GeO–SiO] –2H_2_ and K[GeO–SiO] –2H_2_ (Table 5).

## Conclusion

In this paper, the adsorption behavior of H_2_ molecules on the Li[GeO–SiO], Na[GeO–SiO] or K[GeO–SiO] surfaces are systematically studied by first-principles calculations. H-grabbing by Li[GeO–SiO], Na[GeO–SiO] or K[GeO–SiO] heterostructure was investigated by first-principles computations of DFT method. The alterations of charge density illustrated a remarkable charge transfer towards Li[GeO–SiO], Na[GeO–SiO] or K[GeO–SiO]. The fluctuation in charge density values demonstrates that the electronic densities were in the boundary of adsorbate/adsorbent atoms during the adsorption status. Besides, thermodynamic parameters describing H-grabbing by alkali metals-based nanoclusters of Li[GeO–SiO], Na[GeO–SiO] and K[GeO–SiO] have been investigated including internal process of the adsorbent–adsorbate system. It is well established that the addition of Li, Na or K to cell batteries may increase the energy storage in cell batteries. In this work, we explore the effect of Li, Na or K on GeO–SiO heterocluster. Moreover, hydrogen bond (H-bond) accepting sites by Li[GeO–SiO], Na[GeO–SiO] or K[GeO–SiO] can alleviate parasitic hydrogen evolution in aqueous electrolytes in lithium, sodium, or potassium-ion batteries. Notably, at the same levels of grabbing, the K/Na-capture framework exhibited the strongest H_2_ binding.

## Data Availability

No datasets were generated or analysed during the current study.
